# Supplementary material for: Learning curve and surgical outcome of robotic assisted colorectal surgery with ERAS program
Source: Sci Rep. 2022 Nov 29;12:20566. doi: 10.1038/s41598-022-24665-w (PMC9709162; doi:10.1038/s41598-022-24665-w)
Supplement: Supplementary file 2 — Supplementary Information 2. [file 41598_2022_24665_MOESM2_ESM.zip › RAL Raw data-Ñ[▒K/099/HS_ñw╝╨░O▒Kñσ.pdf]

正式摘要(電子簽章：000000221047236)<br>W72 / W72 ☐英文標題(注意:無英文姓名-請補病人基本檔!)	☐【讀取】憑證	

臺中榮民總醫院

出院病歷摘要

轉入號： 轉出號：

[redacted]

(8)流水編號	
(9)入院日期	110/07/16 (2021) 直腸外科 W72 - 102
(10)轉科日期	
(11)出院日期	110/07/20 (2021) 住院天數計 4 日

診斷

- 入院 :1. Rectal adenocarcinoma, 15cm FAV, with bleeding and bloody stool, s/p colonscopic biopsy on 2021/6/25
  2. History of CAD, basal lateral wall ischemia (by myocardial perfusion scan in 2017) (latest 2D-echo on 2021/06/23: LVEF 54%, normal LV wall motion.); under Bokey
  3. Arrythmia
  4. Delirium, with altered congition
  5. Polyneuropathy with neuropathic pain s/p TCA
  6. Dysphagia and dysarthria, suspected intracranial lesion, suspected medication related s/p speech therapy
  7. HTN, under Olmesartan and Urief(?)
  8. Newly-diagnosed type 2 DM, HbA1c 6.6%
  9. Bacteremia with group B streptococcus, s/p tazocin (2021/6/20-6/24), then amcipicillin
  10. Bilateral OA knee, s/p bilateral TKA
  11. L-spine injury, s/p VP
- 出院 :1. Rectal adenocarcinoma, 15cm FAV, with bleeding and bloody stool, s/p colonscopic biopsy on 2021/6/25, s/p Robotic LAR on 2021/07/17
  2. History of CAD, basal lateral wall ischemia (by myocardial perfusion scan in 2017) (latest 2D-echo on 2021/06/23: LVEF 54%, normal LV wall motion.); under Bokey
  3. Arrythmia
  4. Delirium, with altered congition
  5. Polyneuropathy with neuropathic pain s/p TCA
  6. Dysphagia and dysarthria, suspected intracranial lesion, suspected medication related s/p speech therapy
  7. HTN, under Olmesartan and Urief(?)
  8. Newly-diagnosed type 2 DM, HbA1c 6.6%
  9. Bacteremia with group B streptococcus, s/p tazocin (2021/6/20-6/24), then amcipicillin
  10. Bilateral OA knee, s/p bilateral TKA
  11. L-spine injury, s/p VP

主訴

- Tarry and bloody stool occurred on 6/23 and 6/25

病史

- This 86-year-old man has past history of:
  1. Rectal adenocarcinoma, 15cm FAV, with bleeding and bloody stool, s/p colonscopic biopsy on 2021/6/25
  2. History of CAD, basal lateral wall ischemia (by myocardial perfusion scan in 2017) (latest 2D-echo on 2021/06/23: LVEF 54%, normal LV wall motion.); under Bokey
  3. Arrythmia
  4. Delirium, with altered congition
  5. Polyneuropathy with neuropathic pain s/p TCA
  6. Dysphagia and dysarthria, suspected intracranial lesion, suspected medication related s/p speech therapy
  7. HTN, under Olmesartan and Urief(?)
  8. Newly-diagnosed type 2 DM, HbA1c 6.6%
  9. Bacteremia with group B streptococcus, s/p tazocin (2021/6/20-6/24), then amcipicillin
  10. Bilateral OA knee, s/p bilateral TKA
  11. L-spine injury, s/p VP

  During last admission at GERI ward for change of altered mental status, tarry and bloody stool occurred on 6/23 and 6/25. The LGI scope reported rectal cancer with bleleding s/p biopsy on 6/25. CRS was consulted, and the risk of surgery was explained. CEA was 4.62ng/ml, CA-199 was 11.60 Unit/ml. Abdominal CT was done on 2021/06/21. PET was arranged on 7/14, witht pending report.
  After discussion, the operation was scheduled.
  Under the impression of Rectal adenocarcinoma, 15cm FAV, he was admitted for Robotic LAR scheduled on 2021/07/17.

體檢發現

-
  - General appearance :
    - easy looking , conscious clear , without cardiopulmonary distress
    - Vital signs自動帶入date 體溫(BT) 脈搏(P) 呼吸(R) 血壓(BP) 身高(BH) 體重(BW) BMI
      20210716 35.8C 77/min 16/min 124/65mmHg 159.2cm 63.9kg 25.2
    - Vital signs: B.P. 124/65 mmHg , P.R. 77 /min , R.R. 16 /min , B.T. 35.8 ℃
  - HEENT :
    - Eyes: no anemic conjunctiva, no icteric sclera, normal EOM
    - ENT: no otorrhea, no rhinorrhea, no erythematous mucosa, no injected tonsil, no deformity
  - Neck: supple, freely movable, trachea midline,no deformity, no engorged jugular vein , no palpable thyroid or lymph node enlargement
  - Chest:
    - Inspection: Grossly normal, symmetric and fully expansion, no accessory muscle used, no operation scar, no spider angiomata, no gynecomastia
    - Palpation: no palpable mass or nodule, no subcutaneous emphysema, no tenderness
    - Ausculation: clear breathing sound
  - Heart:
    - Palpation: no thrill, no heave
    - Percussion: normal heart size
    - Ausculation: regular heart beat, normal S1, S2, no gallop, no murmur
  - Extremities: No deformity, full ranged of motion without limitation, no clubbing finger or nail cyanosis, no pitting edema, no ulcer or pigmentation, no obvious skin lesion, no varicose vein, no bone, joint or muscle tenderness

CRS CONDITION

- Karnofsky performance status: (100) Normal, no complaints, no evidence of disease
- PE of Abdomen:
- __1. Inspection: flat
- __2. Ausculation: normoactive
- __3. Palapation: non-tender
- CEA: 4.62 ng/ml
- Imaging Studies:
- Colonoscope report: rectal cancer with bleleding s/p biopsy on 6/25.
- Figures:
  1. colon & rectum

手術日期及方法

- 手術日期及方法20210717 1. Robotic (XI) assisted low anterior resection
  2. Intraoperative colonoscopy : (+)
  3. High ligation (+) : At IMA root (Via robotic clips). IMV was ligated via endoclip.
  4. Take down from the splenic flexure : (-)
  5. Robotic EndoGIA (45-4.3) X2 + EEA (29mm) were applied.
  6. Proximal cut end: > 10 cm, distal cut end: 3 cm
  7. No drainage tube was inserted
  8. ADHESION BARRIER FILM was placed

住院治療經過

- After operation with Robotic LAR on 2021/07/17, the patient was under post-operative care and pain control under PCA use. Self-urination was smoothly after removal of Foley's catheter on 7/18. The patient tolerated clear liquid diet and then Bland No.II diet since 7/19, without N/V, abdominal pain or discomfort. The wound was clean and dry, without infectious signs. Under relative stable condition, the patient was discharge with OPD follow up.

合併症

一般檢查

- 累積報告 :

DATE	TIME	ALB	TP	BIL.T	BIL.D	ALKP	AST	ALT	LDH	AMY	LIPASE	NH3	Ferritin	UA
1100620	2318	3.6		1.15			28	25						
1100623	0826	3.5		1.05	0.34	184	36	45						
1100625	1723		5.7											
1100706	1302	3.9		0.67	0.19	132	19	17	146					
1100717	1408	3.6		1.13			16	11						
1100720	0846			0.8			15	8						

DATE	TIME	WBC	RBC	HGB	HCT	MCV	PLT	BLST	NEUT	BAND	SEG	LYM	MON	EOS	BAS
1100620	2318	18170		12.3			278		87.6			5.1	7.2	0.0	0.1
1100623	0829	9190	3.79	11.4	33.4	88.1	327		76.3			11.2	8.7	3.6	0.2

1100624	0828	6530		10.9			287		70.4			15.0	10.3	4.0	0.3
1100706	1302	8370	3.76	11.8	34.9	92.8	270		73.8			17.7	6.3	1.7	0.5
1100717	1408	8070		11.8	36.1		169		91.3			7.8	0.7	0.1	0.1
1100720	0856	5050		10.1	30.6	93.3	155		66.4			17.4	9.1	6.7	0.4

DATE	TIME	NA	K	CL	BUN	CREAT	eGFR	CA	Mg	P	CRP	ESR	PCT	D-Dimer	LACTATE
1100620	2318	119	4.5	86	35	0.94	80.88	9.1			29.823				10.7
1100621	0539														7.3
1100621	0540	122											0.233		
1100623	0826	128	4.0		23	0.91	83.96	8.5			9.312				
1100624	0821														10.5
1100624	0822	129	4.0												
1100624	0832													1.47	
1100706	1302	135	5.1		22	1.18		9.1							
1100717	1408	135	3.9		12	0.94		9.1							
1100720	0846	136	3.9		7	1.03	72.78								

DATE	TIME	GLUCOSE	HbA1C	CHOL	TG	HDL	LDL	KETONE	CK	CKMB	TRO-T	PT-P	APTT-P	NT-pro BNP
1100620	2318	148							73		31.23	11.0	33.1	
1100623	0829		6.6											
1100624	0832											10.8	29.1	
1100625	1358											11.3	31.6	
1100706	1302	129										10.6	28.3	

DATE	TIME	SP-GR	PH	PROT	GLUC	KETON	BILI	UROB	OB	NITR	LEU	RBC	WBC	EPCEL
1100621	0036	1.030	6.0	3+	>1000.0	Negative	Negative	1.0	Negative	Negative	Negative	0-2	0-2	1-2
1100622	0027	>1.040	6.0	2+	>1000.0	Negative	Negative	1.0	Negative	Negative	Negative	0-2	0-2	0-1

DATE	TIME	PH	PCO2	PO2	SO2	BEB	HCO3	TCO2	HGB	FIO2
1100620	2319	7.378	36.8	45.5	79.7	-3.3	21.2	22.4		
1100621	0540	7.310	53.6	29.0	47.8	-0.8	26.4	28.0		

DATE	TIME	PH	BEB	SCVO2	PO2	HCO3	HGB	PCO2	TCO2
1100624	0855	7.372	1.6	52.1	28.7	27.7		48.8	29.2

特殊檢查

病理報告

其它

出院時情況

- 3.改門診治療

出院指示

藥名	劑量	劑量單位	用法	頻次	數量	數量單位	現況	天數	單位
Ultracet tab	1	TAB	PO	Q6H	28	TAB	TH	7	天
Celebrex cap 200mg	1	CAP	PO	QDPRN	7	CAP	TH	7	天
Through tab	1	TAB	PO	QN	7	TAB	TH	7	天
Mag. Oxide tab	2	TAB	PO	BID	28	TAB	TH	7	天
(□)Takepron OD tab 30mg	1	TAB	PO	QDAC	7	TAB	TH	7	天

出院計劃

- 門診日期
  - date on 2021/07/27

主治醫師_2062F 蔣鋒帆_____ / 住院醫師_2345B 陳映晴_____

正式摘要(電子簽章：000000221047236)

W72 / W72

doctorRank:B
